# Generational differences in international research collaboration: A bibliometric study of Norwegian University staff

**DOI:** 10.1371/journal.pone.0260239

**Published:** 2021-11-29

**Authors:** Kristoffer Rørstad, Dag W. Aksnes, Fredrik Niclas Piro

**Affiliations:** Nordic Institute for Studies in Innovation, Research and Education, Oslo, Norway; Georgia Institute of Technology, UNITED STATES

## Abstract

This paper addresses the relationship between age and international research collaboration. The main research question is: do younger researchers collaborate more internationally than their senior colleagues? A common assumption is that younger generations are generally more internationally oriented than older generations. On the other hand, senior researchers may have larger international networks compared to younger colleagues. The study is based on data for 5,600 Norwegian researchers and their publication output during a three-year period (44,000 publications). Two indicators for international collaboration are used: The share of researchers involved in international collaboration measured by co-authorship and the average proportion of publications with international collaboration per researcher. These indicators reflect two different dimensions of international collaboration. Although the findings are not consistent across age cohorts and indicators of internationalization, the overall trend is that international collaboration tends to decline with increasing age. This holds both at aggregate levels and within groups of academic positions. However, the generational differences are not very large, and other variables such as the field of research explain more of the differences observed at an individual level.

## Introduction

The extent of international research collaboration has increased significantly in recent decades [1, 2]. In many countries, the majority of scientific publications are now internationally co-authored. In Norway, this proportion has grown from approximately 20 per cent during the 1980s to more than 60 per cent in recent years (Web of Science indexed publications). There are many plausible mechanisms that may explain this development. For example, new information and communication technologies, reduction in transportation costs and more funding being competitive (often relying on high degrees of specialization and/or requirements for international collaboration) have been suggested [3]. However, the inclination to engage in international collaboration may not only result from such factors. Generational differences [4, 5] in “career opportunities and academic norms” [6] may also shape different practices across generations. Here, we study international research collaboration as such a practice. *In the present study we compare international collaboration rates across age groups* using co-authorship data for Norwegian university researchers in the period 2015–2017. *The aim of the study* is to investigate whether younger and older researchers are differently engaged in international research collaboration.

### International collaboration is integral to evaluations and funding mechanisms

From a policy perspective, and certainly within the individual higher education institutions themselves, the understanding of how different generations contribute to internationalization of research is important. Bibliometric indicators play an (increasingly) important role in most European countries either in research evaluations or in performance-based funding systems at national levels [7, 8]. Here, international collaboration is an important intermediate factor in the individual characteristics of the publication output/impact dimensions [6], as well as being a ‘success indicator’ in itself. The importance of international cooperation in relation to publication output and citation impact (which are typical performance indicators in evaluations and performance-based funding) can be explained along two axes. First, it has *empirically* been demonstrated that so-called international papers (i.e., papers with authors from more than one country) generally are more cited than national papers [1], especially for publications from developing countries [e.g., 9]. Several studies also show that researchers who engage in international research collaboration are more productive than researchers who do not [10, 11]. Second, there are several *theoretically* plausible reasons to assume that engaging in international research collaboration is beneficial beyond what can be measured by bibliometric indicators. Such factors may be considered as intermediary steps towards higher productivity and research quality, due to expansion of networks, knowledge sharing, and coverage of necessary academic diversity needed for both publications and proposals (prospects for external funding).

### Limitations in the current literature on international collaboration

Studying how international collaboration varies by age is not new (see overview of former studies in the next section). Although international research collaboration may take many forms, and scientific publishing may not necessarily be one of them, studying *scientific publications* is the common approach in studies of research collaboration [6], despite its limitations as a measure of collaboration [12]. Here, publications with co-authors affiliated with institutions in different countries (author addresses) are used to measure international collaboration. We also base our study on scientific publications, but believe our study has three main advantages compared to the current literature.

*First*, most studies with a bibliometric approach rely upon data from the Web of Science or Scopus. The poor coverage of Social Sciences and Humanities in these databases is well known [13]. In our study, we use a national Norwegian publication database, covering all researchers in Norway and all fields, where monographs and anthologies are registered on similar terms as journal articles.

*Second*, unlike former studies that have been restricted to certain academic positions, our study includes all researchers from the youngest doctoral students to the oldest professors.

*Third*, studies differ in whether they measure international collaboration according to whether they have collaborated internationally or not (a binary response) or whether they measure the degree of international collaborations (a continuous variable, typically expressed as percentages or ratios). In our study we will present results for both measures, thus eliminating the chance of presenting results for one of the two indicators, which may be reversed for the other.

*Finally*, unlike the survey approach often being used in non-publication-based studies, where respondents’ claims about international cooperation are self-reported and with inevitable questions about content validity, we rely upon verifiable publication data from a total population of researchers with no selection bias or validity concerns. Previous studies on the relation between age and international collaboration have not combined the entire spectrum of age groups, all scientific and scholarly fields, and publications in a similar manner to our study, presenting both binary and continuous measures for international collaboration.

### Age differences in academia

The literature on how different attributes of researchers (such as gender, academic position and age) are associated with bibliometric performance measures (foremost publication and citation indicators) is extensive. The focus of this study is the age dimension, where many previous studies have investigated how the age of scientists influences on their scientific performance. In particular, the issue has been addressed in relation to publication productivity. The findings from these studies are not entirely consistent, but it seems to be quite firmly established that there is a curvilinear relationship between age and *productivity*. The average production of publication increases with age and reaches a peak at some point during the career and then declines [see e.g., 14–16]. As for age and *citation impact*, some studies have found, in line with Merton & Zuckerman’s [17] claim of science as a “young man’s game”, that citation impact is diminishing with increasing age [e.g., 18]. Others have found a decline of (average) citation impact until a certain age [19]. For example, Gingras et al. [15], identifying the age of 50 years old as the turning point (while the production of highly cited papers kept rising continuously until retirement). A more explorative approach has been to study academics’ *creativity*, such as Liu et al. [20] who found creativity to be spread across certain time intervals of a career, where for most researchers it is a matter of having one such ‘hot streak’, while Jones et al. [21] concluded that great scientific output (based on awards and prices) typically peaks in middle age. Related to the latter, is Gingras et al.’s [15] study of Canadian university professors showing a ‘stagnation’ in their ability or willingness to pick up on new ideas. Here, at the age of 40, there was a slow-down in productivity and a less ambitious approach to citing literature (the professors kept relying on the older literature, i.e., they became less inclined to pick up on new literature). The second turning point was at age 50, where the productivity was at its highest, while the citation impact was at its lowest. At this stage the professors had begun moving from first author positions towards the end of the author list. The above-mentioned studies (and many more) clearly support the notion of *age* playing a part in both preferences, processes and outcomes of academics: research performance may improve/increase or worsen/diminish (or quite simply change) by age, or it may be accentuated at certain points during the career. There are different theoretical models that may be relevant for explaining why one might expect generational differences in international collaboration, with two main contrasting lines of research. One emphasizes changes in individual behavior (i.e., globalization increasingly stimulating young researchers), while another line of research focuses on structural changes in the science system (i.e., favoring the older researchers).

### Young researchers are a product of a more international era

The behaviors of individuals are influenced by the generation they belong to [7, 22]. This means that younger and older scientists may deviate in their research practice due to differences in the socio-cultural influences at different times. Generational differences may be seen because researchers have been trained and socialized into the academic profession in divergent ways (4); the older researchers were trained at a time when international collaboration was much less common than today. Abramo, D’Angelo & Solazzi’s [3] claim that “the increasing costs of research and the complexity of certain undertakings prevent individual researchers, institutions and even nations from taking on certain themes alone (either completely or in an efficient manner)”. Thus, it is reasonable to suggest that young researchers to a much higher extent have been trained in R&D systems with coerced international collaborations.

Several studies have pointed at an “age-creativity relationship” [23], claiming that younger researchers are more open to new ideas [24]. Moving beyond the STI literature (science and technology indicators), such reasoning is well aligned with theories of globalization and cosmopolitan identity predicting that younger people have more global identities [25], where e.g. Stohl & Ganesh [26] describe how globalization has undergone three key phases: from the ‘generation of uncertainty’, to the ‘generation of connectivity’ where increased interdependency and complexity necessitated cooperation (with heavy transaction costs), to the ‘generation of ubiquity’ where transaction costs are heavily reduced and being global is just routine behavior.

The increasing internationalization of higher education and international mobility of researchers, and perhaps even more importantly, students in their younger and formative years, may be stimulating to the willingness for future international collaborations [27, 28]. But the push towards internationalization may also be externally encouraged. In line with institutional strategies and goals, younger researchers today are more expected to be (or even pushed towards) more international work than older generations were [4]. Furthermore, cross-national cooperation may not only be encouraged but also a requirement for research funding, which is clearly seen in many funding schemes, foremost in the European Framework Programs for Research and Innovation.

Elaborating on ‘utility maximizing theory’, Kwiek [4] makes arguments about the incentives of researchers for engaging in activities such as international work and publishing. With reference to Kyvik [29], he suggests a (theoretically) diminishing willingness of older researchers to pick up on those activities that at a younger age were considered advantageous, foremost with regards to job promotion: increasing one’s academic production, increasing one’s reputation, etc. Based on such arguments, we may argue that the instrumental motivation of younger researchers to engage in international (funding and publication promoting) cooperation is higher than for older researchers [see also 30, 31]. Furthermore, for older researchers more leadership and administrative work loads may reduce the maintenance of international networks and research activities [32, 33]. Hence, generational differences favoring the young may result from both ‘tales’: both an increased tendency towards internationalization among the young and a decreased tendency among the older.

Against this background, based on a dataset of Norwegian researchers, we will assess our *main hypotheses* and possible explanations: *Younger researchers are more internationally oriented than their senior colleagues are*.

### Preferential attachments–Favoring the older generations

Despite generational differences plausibly leading younger researchers towards more international collaboration, both empirical and theoretical arguments for a reversed association are easy to find, i.e., the alternative hypothesis: *Older researchers are more internationally oriented than their younger colleagues are*.

A plausible explanation for older researchers’ potentially higher degree of international cooperation, is related to the possibility for external funding [34], which may be dependent on past productivity [35]. With increasingly more funding coming from competitive external sources, the second hypothesis would be supported by findings from the United States where there has been a steady rise in the age at which US researchers receive their first grants [15, 36]. Funding is important in order to create research groups, and the work of Ebadi & Schiffauerova [37] describes how research networks usually are led by a core of productive researchers, while younger researchers are involved in more mediatory positions in the networks.

Several studies investigating Italian data have found a correspondence between cooperation and productivity [3, 38], and academic position [32]. The latter study did, however, not include the full age-spectrum, as it was restricted to full, associate and assistant professors. In a survey of 11 European countries, Kwiek [6] found strong generational differences in international research cooperation: in most countries studied, it was the older and oldest generations that were the most international. The only exceptions were Netherlands, Switzerland and Austria, where the youngest generations were more international.

To some extent, we may describe older researchers’ international collaborations of today as driven by the past. There is a certain ‘Matthew effect’ pertaining to this: as a researcher’s curriculum vitae expands over the years based on number of publications, number of grants received and projects led, and the attractiveness of the researcher as a collaborating partner grows. Working in the opposite direction, from the outset of the researcher, is the self-organized willingness to engage in international collaboration that may also increase the level of internationalization, cf. an ever increasing and continuous search for recognition, rewards and visibility, so-called ‘preferential attachment’ [39], where prolific researchers over time become increasingly more central nodes in research networks. Hence, researchers with larger research grants (typically group leaders, and older and merited researchers) tend to collaborate more internationally [40]. Collaborating internationally may not only be a result of a person’s willingness to do so, but also conditional on the person’s ability to do so, which may be depending on factors such as reputation, networks and funding possibilities.

As showed by the discussion above, the previous findings on the relation between age and international collaboration are not entirely consistent. There are also studies showing less of a gradient, but more complex relationships. For example, in a Norwegian study where academic staff were asked to report if they had collaborated with foreign colleagues during a three-year period [41], the propensity to collaborate internationally were lowest for the youngest and oldest age-groups (below 35 years old and above 60) and similar findings were reported in an updated survey [7, 42]. Such findings are supported by studies of how researchers’ publishing practices changes over time [15].

## Data

In the present study, we are analyzing the issue of generational differences in international research collaboration using bibliometric data. The study is based on a dataset consisting of 5,554 Norwegian researchers at the four largest traditional universities in Norway: University of Oslo, University of Bergen, The Arctic University of Norway and The Norwegian University of Science and Technology. The study sample includes professors (full-professors), associate professors, postdocs and PhD-candidates with at least one publication during the years 2015–2017 (43,641 publications).

The study is based on a Norwegian bibliographic database called *Cristin*. This is a current research information database used for storing and managing publication metadata, as well as for the purpose of performance-based funding of the institutions [43]. The database is applied by all public research institutions in Norway. In the database, all peer-reviewed scientific and scholarly publication are indexed, including books, articles in edited volumes and conference series. There are particular requirements and criteria concerning what type of publications that can be approved as scientific/scholarly. Moreover, lists of approved journals and publishers are revised annually by expert-panels in each discipline (for further information see [43]). Based on these principles, the database has a complete coverage of the scientific and scholarly publication output of Norwegian researchers. Compared with Web of Science, an earlier study showed that it contains 45 per cent additional publications [13].

The database contains structured data which can be applied for bibliometric analysis. For example, all authors and addresses are indexed, including country as a controlled term, which is important for the study of international collaboration. All publications are attributed to individuals and these data are verified by the authors and institutions. Thus, there is no need for author disambiguation, which is often a problem in studies based on other databases [44]. In the Cristin database, all publications are classified into five broad areas (engineering and technology, humanities, medical and health sciences, natural sciences, and social sciences) and 85 subfields/disciplines. We have assigned the individuals to the broad areas and subfields/disciplines in which they had the largest number of publications. In some cases, an individual had published an equal number of publications in two or more categories. These individuals were randomly distributed to one of these categories.

In addition, we have used the *Norwegian Research Personnel Register* as a supplementary data source of the individual attributes of the researchers analyzed (institution, position, gender and age). The individuals were classified in 10-year age cohorts defined as the aggregate of individuals who were born in the same time interval. Although we might lose some of the data variation, age intervals simplify the descriptive presentation and interpretation at an aggregated level. However, in the regression analysis, we use age as a continuous variable.

The distribution of researchers by academic position, fields and age groups is shown in Table 1.

**Table 1:** Distribution of number of researchers by major fields, position and age groups (N = 5,554).

*Position/fields*	*Below 30 years*	*30–39 years*	*40–49 years*	*50–59 years*	*Over 60 years*	*Total*
Professors		49	484	777	758	2,068
*Humanities*		*10*	*81*	*124*	*129*	*344*
*Social sciences*		*13*	*90*	*155*	*170*	*428*
*Natural sciences*		*10*	*129*	*175*	*161*	*475*
*Engineering and technology*		*10*	*82*	*96*	*73*	*261*
*Medical and health sciences*		*6*	*102*	*227*	*225*	*560*
Associate professors	3	292	559	312	179	1,345
*Humanities*		*47*	*111*	*67*	*40*	*265*
*Social sciences*		*68*	*149*	*86*	*50*	*353*
*Natural sciences*	*1*	*55*	*104*	*43*	*23*	*226*
*Engineering and technology*	*2*	*58*	*50*	*32*	*14*	*156*
*Medical and health sciences*		*64*	*145*	*84*	*52*	*345*
Postdocs	30	523	100	16	4	673
*Humanities*		*36*	*18*	*2*		*56*
*Social sciences*		*52*	*17*	*2*		*71*
*Natural sciences*	*12*	*198*	*17*	*2*	*1*	*230*
*Engineering and technology*	*8*	*91*	*4*		*1*	*104*
*Medical and health sciences*	*10*	*146*	*44*	*10*	*2*	*212*
PhD-candidates	625	656	156	27	4	1,468
*Humanities*	*20*	*79*	*17*	*2*		*118*
*Social sciences*	*43*	*97*	*33*	*7*	*3*	*183*
*Natural sciences*	*234*	*133*	*8*	*3*	*1*	*379*
*Engineering and technology*	*207*	*105*	*10*	*2*		*324*
*Medical and health sciences*	*121*	*242*	*88*	*13*		*464*
**Total**	**658**	**1,520**	**1,299**	**1,132**	**945**	**5,554**

The researchers are not equally distributed across positions and age group. Moreover, the distribution of publications differs significantly from the distribution of researchers (see Appendix Table 1 in S1 Appendix). The researchers below 50 years constitute about 63 per cent of the population, while they account for 53 per cent of the publications. About 37 per cent of the population of researchers are over 50 years old, while they account for 43 per cent of the publications, i.e., senior personnel publish on average more than their younger colleagues do. If we compare the balance between academic positions and publication output, professors are the most productive group. The professors account for 37 per cent of the population of researchers and 59 per cent of the publications. In the analyses, we also include the academic position of the researchers. Thus, the analyses are carried out by field of research, academic position, and scientific production.

## Study design and methodology

Two indicators of international collaboration are applied. First, the share of researchers who have been involved in international collaboration measured by co-authorship (i.e., have published at least *one* publication involving international co-authorship). This is a measure of whether the individuals have collaborated internationally or not [cf. 41]. We denote this measure of international collaboration, *indicator A*. Second, the average proportion of the researchers’ publications involving international co-authorship. This is a measure of the intensity of the international collaboration [32]. We denote this measure of international collaboration, *indicator B*. In the regression analysis, indicator A will have value 1 for those researchers who have had international co-authors, or 0 for those who have only had Norwegian co-authors. Indicator B will have continuous values in the range from 0 to 1.

The unit for the analysis is the individual researcher. In other words, all individuals count equally as one unit in the analysis regardless of how many publications they have published. By this, we avoid that the analysis is biased towards highly productive researchers.

As independent variables, we have selected various measures which are available through the databases applied and which potentially may influence on the collaboration patterns. An overview is provided in Table 2. The research question of the study has been approached by descriptive statistical analyses and regression analyses. In the descriptive statistical analyses, we have included three main independent variables: Age interval, major field and academic position. In the regression analyses we have included additional variables which are relevant to take into consideration when analyzing international collaboration at the level of individuals. A further justification for including these variables is given below.

**Table 2:** Overview of the variables applied in the study with descriptive statistics.

**Independent variables**	**Type of variable**	**N**	**Mean**	**Std. Dev**	**Min**	**Max**
Age	Continuous	5,554	44.95	12.45	24	76
Gender	Binary	5,554	0.58	0.49	0	1
Full professor	Categorical/dummy	2,068	0.37	0.48	0	1
Associate professors	Categorical/dummy	1,345	0.24	0.43	0	1
Post Doc	Categorical/dummy	673	0.12	0.33	0	1
PhD-student	Categorical/dummy	1,468	0.26	0.44	0	1
Productivity group 1 (1 pub)	Categorical/dummy	954	0.17	0.38	0	1
Productivity group 2 (2 pub)	Categorical/dummy	798	0.14	0.35	0	1
Productivity group 3 (3–4 pub)	Categorical/dummy	1,161	0.21	0.41	0	1
Productivity group 4 (5–7 pub)	Categorical/dummy	1,004	0.18	0.38	0	1
Productivity group 5 (8–12 pub)	Categorical/dummy	827	0.15	0.36	0	1
Productivity group 6 (13 and more pub)	Categorical/dummy	811	0.15	0.35	0	1
Average numbers of publications	Continuous	5,554	5.52	10.35	1	205.68
**Dependent variables**	**Type of variable**	**N**	**Mean**	**Std. Dev**	**Min**	**Max**
Indicator A (International collaboration, yes or no)	Binary	5,554	0.618	0.49	0	1
Indicator B (average proportion of publications with international collaboration per researcher)	Continuous	3,436	0.35	0.36	0	1

### Scientific field

There are large differences across fields and disciplines in international collaboration rates measured by co-authorship [45, 46]. The proportion of publications involving international collaboration is generally much higher in the natural sciences, engineering and technology and medicine and health sciences, than in the social sciences and humanities. In the humanities, single-authored publications are still most common, which means that this field typically will have low rates of international collaboration [46]. These differences are also observed in the publication output of Norwegian researchers (Table 3). There is also a strong association between collaboration generally (indicated by number of authors per publication and proportion of solo authored publications) and international collaboration. These field differences will have large impact on the results obtained. If not taken into consideration, possibly with strong age differences between them, they may add to misinterpretation of the overall results. We also believe it is important to include those fields where international collaboration/co-authorship is less common as these fields to date are unexplored. This also ensures that our study covers academia in its entirety.

**Table 3:** Overview of basic co-authorship measures across major fields (2015–2017 publications).

Major field	Average number of authors per publication	Proportion solo authored	Proportion with international collaboration	N (publications)
Humanities	1.5	73%	14%	7,914
Social Sciences	2.2	41%	30%	15,069
Natural sciences	10.5	6%	70%	17,299
Engineering and technology	4.1	7%	49%	11,427
Medicine and health sciences	8.6	6%	58%	19,616

### Academic position

Access to international networks may relate to the academic rank of the researchers, cf. theory of preferential attachments and the Matthew effect. This also reasons with former studies finding that productivity increases within the hierarchy of academic positions.

### Gender

Many previous studies have shown that there are large gender differences in productivity where female researchers on average publish fewer publications than their male colleagues [47]. Gender differences have also been observed for international research collaboration, but here the findings are less consistent. In a study of gender differences in international collaboration, using the same dataset as in the current study, it was concluded that when field, academic rank, and productivity were taken into consideration, there were only minor and non-statistically significant gender differences in international collaboration rates (48). Because men and women are differently distributed across academia by age (more women are found in the younger age groups), we include gender as a control variable, not to overestimate age effects potentially originating from skewed gender distributions.

### Scientific productivity

Productivity is skewed at the level of individuals. A large part of the researchers has very few publications, while a minority is highly prolific. This issue is important to consider when studying differences in international collaboration between age groups. Previous studies [e.g., 48] have shown that prolific researchers are more likely to collaborate internationally then non-prolific researchers. For this reason, we have taken productivity into account in the regression analysis.

The researchers have been classified into six groups based on their publication output during the period. The groups are approximately equally sized regarding numbers of researchers in each group. Group 1 includes researchers with only 1 publication (17 per cent of the researchers). Group 2 is researchers with two publications (14 per cent). Group 3 is researchers with three and four publications (21 per cent). Group 4 is researchers with five to seven publications (18 per cent). Group 5 is researchers with 8 to 12 publications (15 per cent), and group 6 includes researchers with more than 12 publications and account for 15 per cent of the researchers in total. Group 1 and 2 can be categorized as researchers with low production, while group 3 to 5 can be considered intermediate productive, while group 6 is the most prolific researchers. By this classification we will be able to provide further insight into how international cooperation relates to productivity.

### Average number of authors per publication for each individual

Previous studies have shown that researchers involved in international collaboration tend to publish papers with more co-authors than other researchers [48]. However, there are large field differences in the number of co-authors, with large-scale international infrastructure projects such as CERN as the extreme example. The likelihood of collaboration internationally, increases for researchers with many co-authors. This variable is therefore included in our analysis.

### Regression analysis

In addition to descriptive statistical analyses, we have conducted regression analyses on both dependent indicators: researchers that collaborates internationally or not (A) and their proportion of papers with international co-authors (B). The purpose of the regression analyzes is to see whether there is an independent association between age and international collaboration, taking into account other compositional factors that have been demonstrated in former studies to influence on international collaboration. A negative correlation between age and the two dependent variables will tell if the degree of international collaboration decreases with increasing age. For indicator A, every researcher is assigned a value of either 0 or 1, where 1 means that the researcher has collaborated internationally and 0 means that he/she has not. For this binary indicator, we have conducted a logistic regression analysis. For indicator B (proportion of international collaboration), we conducted an ordinary least squares linear regression, where the dependent variable (degree of international collaboration varying continuously from 0 to 1) was log transformed, due to the lack of normal distributed data. Because of this transformation, all researchers without international collaboration were excluded from the analysis (since log of 0 is undefined). Hence, the analysis of indicator B includes only researchers who have collaborated internationally. This implies that we are measuring how age is correlated to international collaboration for researchers who are internationally collaborative.

Since researchers in different scientific fields have different publication patterns, we have analysed this issue separately by major fields. For the regression analyses, the variables have been recoded: all academic positions were recoded to categorical variables and then to dummy variables (1 if present and 0 if not present); the gender variable recoded in the same way (men = 1, women = 0).

## Results

**Fig 1:**
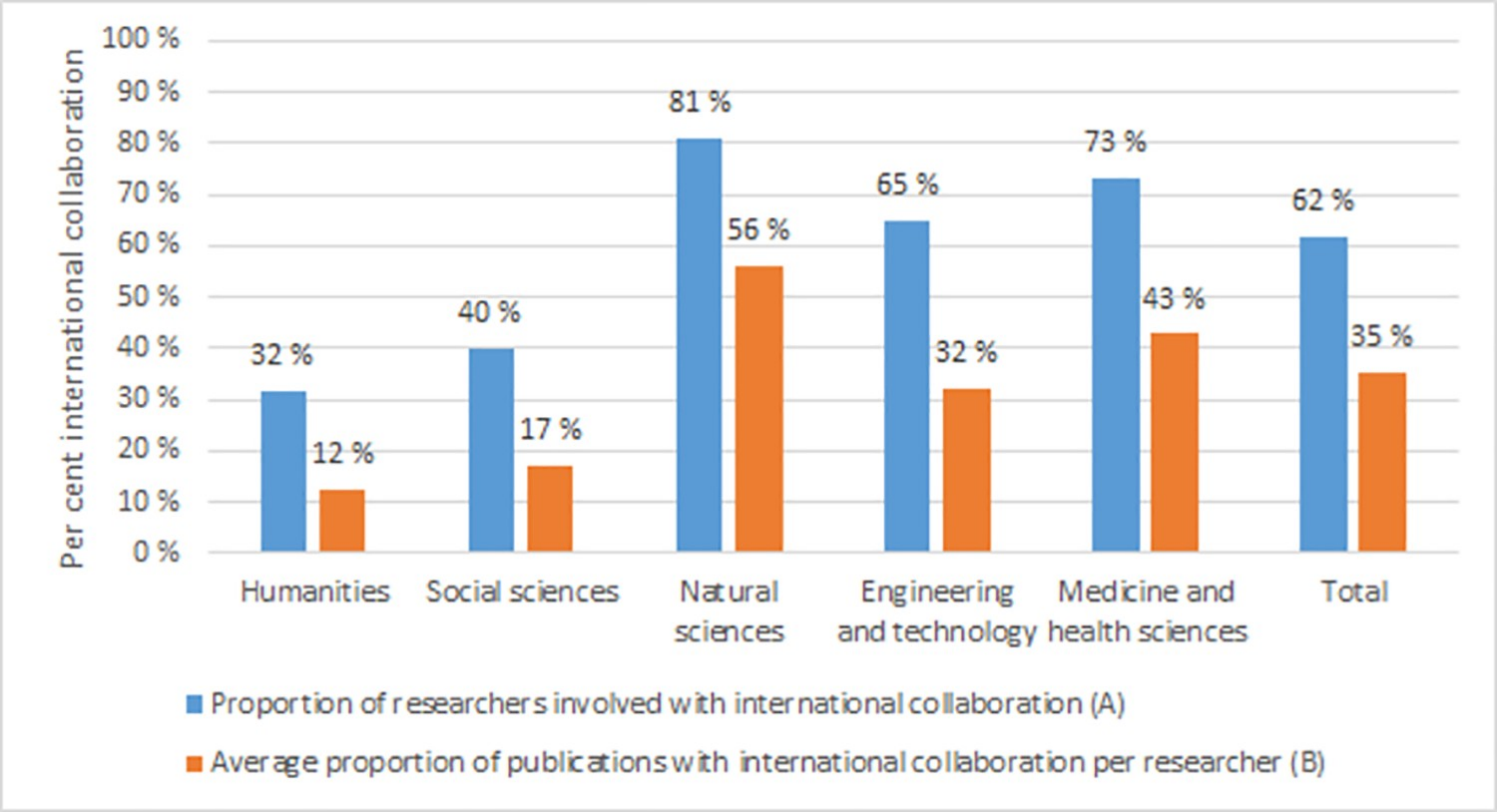
Proportion of researchers involved in international collaboration (A) and average proportion of publications with international collaboration per researcher (B) by major fields.

There are large differences in international collaboration across fields: it is most common in the natural sciences and in medicine and health sciences, and at its lowest in social sciences and humanities (Fig 1). Overall, 62 per cent of the researchers had contributed to at least one international publication over a three-year period (indicator A). For the second indicator, measuring the degree of collaboration (i.e., the share of publications with international co-authorship), we find that among those who did publish internationally, 35 per cent on average of their publications were produced in collaboration with international co-authors (indicator B). For all fields, the proportion of researchers who are involved in international collaboration (A), is higher than the degree of international collaboration (B). This is not surprising as it suffices to have published one publication with a foreign co-author to count as an internationally collaborative researcher.

**Fig 2:**
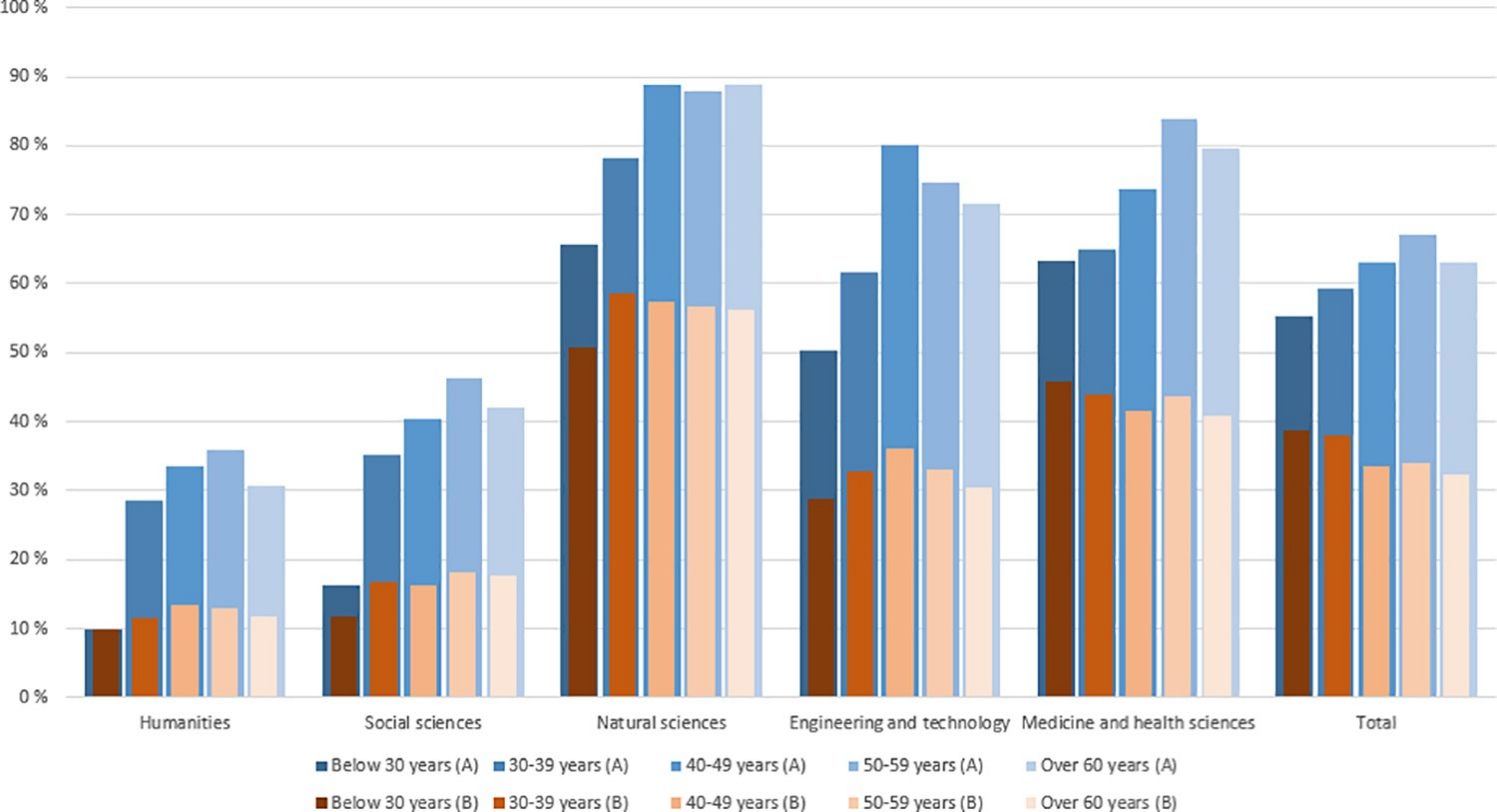
Proportion of researchers (indicator A) involved in international collaboration and average proportion of publications with international collaboration per researchers (indicator B) by major fields and age groups (N = 5,554).

Fig 2 show the proportions of international collaboration measured by both indicators (A and B) by major fields and age groups. Overall, the generational differences are larger on indicator A than B. Indicator A shows that the younger researchers less often have collaborated internationally than older researchers. For Indicator B, the pattern is opposite overall (total), but the difference is not very large and there are variations at field levels.

When we compare international collaboration by age and academic position, we also observe generational differences. Overall (total) the two indicators still show a contrary pattern, but within each position, except postdocs, also Indicator A displays that the youngest researchers are more internationally collaborative then the older ones.

For the full professors, associate professors and PhD-candidates, the propensity to collaborate internationally (indicator A) is declining with age, albeit with some variations in the age patterns. For instance, 78 per cent of the full professors in the age group 30–39 years were involved in international collaboration, while the corresponding figure for professors above 60 years was 66 per cent. For the postdocs, the proportion is highest in the 30–39 age group, and then declines with age.

**Fig 3:**
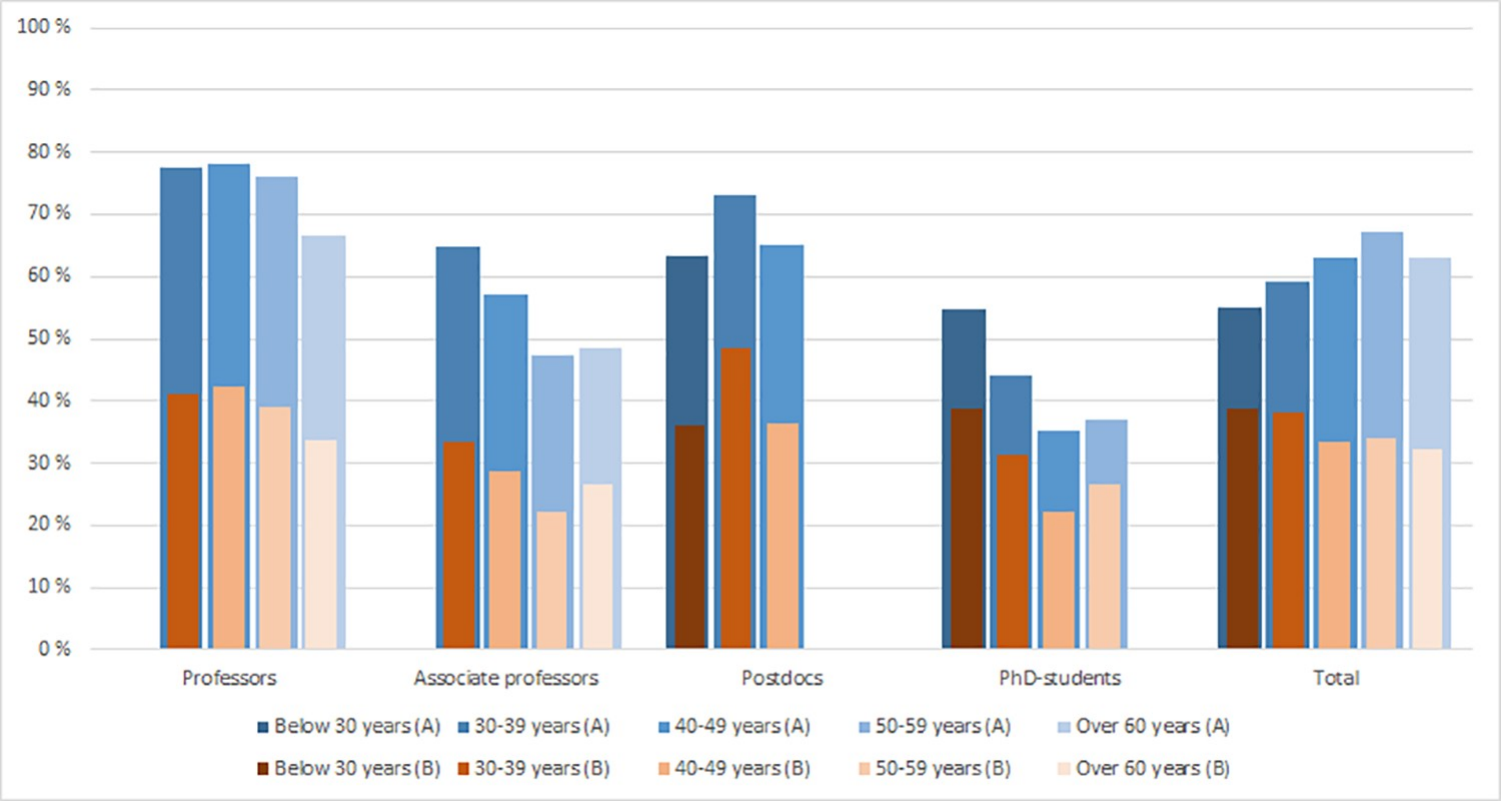
Proportion researchers involved in international collaboration (indicator A) and average proportion of publications with international collaboration per researchers (indicator B) by academic positions and age groups (A) (N = 5,554)*. *) Figures are not shown for categories with less than twenty researchers.

As noted above, the total bar charts in Fig 3 (including all positions) show an opposite age pattern. This might be explained by the Simpson’s paradox [49], which in our case relates to the different composition of researchers across academic positions. PhD candidates, who have the lowest rate of international collaboration, account for the large majority of the individuals below 30 years, while full professors are over-represented in the older age groups.

The alternative indicator (B) measuring the intensity of the international collaboration also shows that the rates of international collaboration generally tend to decline with age. The pattern is most distinct for the full professors and associated professors, while the post docs show a more heterogeneous picture.

We have included additional descriptive statistics in tables in the (S1 Appendix). These tables summarize the statistics for indicator A (Appendix Table 2 in S1 Appendix) and B (Appendix Table 3 in S1 Appendix) by fields and academic positions and age groups at the same time. Looking at full professors only, the younger ones seem to be more international oriented than older professors, and this pattern seems to be valid for several fields on both indicators. For instance, a higher proportion of professors in the age group 40–49 have collaborated internationally than professors over 60 years old, in the same major field, and this applies for all fields (Appendix Table 2 in S1 Appendix).

For all fields, except social sciences, younger professors have on average a higher proportion of their publications with international co-authorship then their older colleagues (indicator B). For the other academic positions, the patterns are mixed. In medical and health sciences, the declining rate of international collaboration applies to all academic positions (Appendix Table 3 in S1 Appendix).

### Regression analysis

We have so far analysed the issue using descriptive statistics combining the two dependent variables with independent variables of age intervals, academic position and major fields. In order to provide further insight into the issue, we have conducted a regression analysis where all variables are considered.

As an initial analysis we calculated the correlation coefficients between all variables included in the regression analyses, except the productivity intervals (Table 4). When considering the indicator B (proportion of internationally co-authored publications), the coefficient is highest for the average number of authors per publication (0.32). As for the age variable, this as expected partly correlated to academic position. The age variable is positively correlated to professors with a value of 0.66 and negatively to PhD-candidates with a value of -0.62 (both statistically significant). The correlation matrix does not identify any strong correlations between the independent variables indicating that there are no collinearity problems. To check for further collinearity, covariance tests for all variables in each field were conducted (Variance of inflation factors, vif-tests in Stata). These tests provided average values for all variables within the fields with average range from 1.85 for humanities to 2.23 for technology and engineering. The largest vif-values found for a variable, where for full professor with values around 4.8 to 6.0, well below the threshold for what must be considered problematic (a rule of thumb is to use the value 10 as an upper threshold) [50].

**Table 4:** Correlation matrix. Average proportion of publications with international collaboration per researcher and selected variables.

Variables	Average proportion of publications with international collaboration per researcher	Age	Gender	Full pro-fessors	Associate professors	Post-docs	PhD-cand.
Average proportion of publications with international collaboration per researcher	1.00						
Age	-0.06*	1.00					
Gender	0.07*	0.12*	1.00				
Full professors	0.06*	0.66*	0.21*	1.00			
Associate professors	-0.11*	0.11*	-0.09*	-0.44*	1.00		
Postdocs	0.11*	-0.28*	-0.04*	-0.29*	-0.21*	1.00	
PhD-candidates	-0.03*	-0.62*	-0.11*	-0.47*	-0.34*	-0.22*	1.00
Average number of authors per publication	0.32*	-0.04*	0.02	0.03	0.05*	0.05*	0.02

Note: Significant correlations within 95 per cent (p<0.05) are marked with *.

### Regression analysis on indicator A

Table 5 shows a summary of the regression analysis for the indicator measuring the proportion of researchers involved in international collaboration (A) for all major fields. The table shows coefficients and standard errors in bracket and stars for statistically significant results for all of the independent variables we have included in the analysis. The table also includes the number of observations and regression coefficients. Since the dependent variable is binary at an individual level, logistic regression analysis was carried out. We have chosen to show the Beta coefficients, rather than odds ratios, for interpretational reasons (i.e., negative age coefficient equals negative correlation to international collaboration).

**Table 5:** Logistic regression analysis. Proportion of researchers involved in international collaboration (indicator A) and selected variables by major fields. Coefficients and std. errors.

Variables	Humanities	Social sciences	Natural sciences	Engineering and technology	Medical and health sciences
Age	-0.029*	-0.012	-0.041**	-0.021	-0.017
	(0.012)	(0.010)	(0.001)	(0.015)	(0.010)
Gender (m = 1, f = 0)	0.116	0.058	0.491*	0.387	0.025
	(0.204)	(0.163)	(0.192)	(0.223)	(0.149)
Full professors	1.239*	1.174***	2.409***	1.388**	0.733*
	(0.505)	(0.350)	(0.479)	(0.471)	(0.316)
Associate professors	1.187**	0.543	1.594***	0.747*	0.758**
	(0.459)	(0.301)	(0.371)	(0.351)	(0.245)
Postdocs	0.644	0.857*	0.902***	0.584	0.531*
	(0.542)	(0.380)	(0.262)	(0.300)	(0.228)
1 publication	-2.139***	-1.604***	-1.770***	-0.770*	-1.294***
	(0.455)	(0.316)	(0.251)	(0.300)	(0.228)
2 publications	-0.1057**	-0.451	-0.672*	-0.522	-0.331
	(0.332)	(0.250)	(0.266)	(0.305)	(0.205)
5 to 7 publications	0.624*	0.970***	-0.476	0.372	1.152***
	(0.254)	(0.212)	(0.283)	(0.256)	(0.227)
8 to 12 publications	1.059***	1.631***	1.778***	1.730***	1.756***
	(0.303)	(0.245)	(0.555)	(0.320)	(0.275)
13 and more publications	2.275***	3.023***	2.627*	2.495***	3.594***
	(0.544)	(0.549)	(1.039)	(0.414)	(0.608)
Average number of authors per publication	1.574***	0.777***	0.578***	0.677***	0.342***
	(0.158)	(0.081)	(0.055)	(0.078)	(0.031)
*Number of observations*	*783*	*1035*	*1310*	*845*	*1581*
*Pseudo R-squared*	*0*.*343*	*0*.*297*	*0*.*382*	*0*.*321*	*0*.*344*

Note 1: Unstandardized regression coefficients, with std. errors in brackets

* p<0.05

** p<0.01

*** p<0.001.

Note 2: Reference variables: PhD candidates and productivity interval 3 (3–4 publications).

Overall, the selected independent variables (age, gender, academic position, productivity, average number of authors per publication) explain 30–38 per cent of the variance dependent on scientific field. The value is lowest for social sciences and highest for the natural Sciences.

We find substantial differences across academic positions where beta coefficients tend to increase by academic rank. This means that higher-position staff are generally more internationally collaborative than PhD-candidates (which is the reference group for the dummy variable). This corresponds with findings presented in Fig 3. At the same time, the more productive a researcher is, he or she is more inclined to have at least one publication with international collaboration. International co-authorship is also correlated with the average numbers of co-authors.

Age of the researchers is negatively correlated to international co-authorship, i.e., younger researchers collaborate more internationally. This holds for all major fields. The coefficient levels are rather low, but here it should be noted that we have used age as continuous variable, and not age groups (10-year intervals). Age is only statistically significant correlated with Indicator A for the natural sciences and humanities. In the other fields, the relationship is also negative, but without significant coefficients.

### Regression analysis on indicator B

The regression analysis of the proportion of internationally co-authored publications (indicator B) shows that the selected variables in total explain 17–37 per cent of the variance (Table 6). The figure is lowest for the Natural sciences (17%), and for the remaining fields the R-square values are in the range of 24% (Medical and health sciences) to 37% (Humanities). The propensity to collaborate with colleagues in other countries can therefore partly be explained by these variables. For all fields, productivity and number of authors per publication are statistically significant variables.

**Table 6:** Linear regression analysis. Average proportion of publications with international collaboration per researcher (indicator B) and selected variables by major fields. Coefficients and std. errors.

Variables	Humanities	Social sciences	Natural sciences	Engineering and technology	Medical and health sciences
Age	-0.002	-0.002	-0.004	-0.009*	-0.008***
	(0.004)	(0.003)	(0.002)	(0.004)	(0.000)
Gender (m = 1, f = 0)	0.080	0.008	-0.045	-0.036	0.052
	(0.069)	(0.058)	(0.034)	(0.064)	(0.032)
Full professors	0.180	0.175	0.079	0.356**	0.132
	(0.169)	(0.138)	(0.071)	(0.121)	(0.070)
Associate professors	0.181	0.182	-0.058	0.018	-0.024
	(0.160)	(0.127)	(0.062)	(0.098)	(0.059)
Postdocs	0.193	0.058	0.048	0.332***	0.030
	(0.194)	(0.151)	(0.049)	(0.089)	(0.053)
1 publication	0.990***	0.811***	0.487***	0.736***	0.639***
	(0.176)	(0.142)	(0.057)	(0.124)	(0.067)
2 publications	0.295*	0.404***	0.121*	0.397**	0.270***
	(0.128)	(0.113)	(0.054)	(0.121)	(0.062)
5 to 7 publications	-0.410***	-0.217*	-0.126*	-0.266**	-0.103*
	(0.092)	(0.085)	(0.050)	(0.091)	(0.005)
8 to 12 publications	-0.427***	-0.451***	-0.086	-0.413***	-0.190***
	(0.102)	(0.087)	(0.049)	(0.091)	(0.053)
13 and more publications	-0.436***	-0.401***	-0.008	-0.335***	-0.135*
	(0.123)	(0.106)	(0.052)	(0.091)	(0.054)
Average number of authors per publication	0.097***	0.089***	0.005***	0.096***	0.013***
	(0.019)	(0.017)	(0.001)	(0.015)	(0.002)
*Number of observations*	*249*	*415*	*1062*	*549*	*1161*
*R-squared*	*0*.*372*	*0*.*328*	*0*.*173*	*0*.*299*	*0*.*236*

Note 1: Unstandardized regression coefficients, with std. errors in brackets

* p<0.05

** p<0.01

*** p<0.001.

Note 2: Reference variables: PhD and productivity interval 3 (3–4 publications).

Since this analysis only includes researchers who have published at least one internationally co-authored publication, the findings will reflect whether the variables contribute to a high degree versus a low degree of international co-authorship. For productivity there is a negative correlation. This might seem counterintuitive and is also in conflict with the results of Table 3. A plausible explanation is that for researchers with low production (1 and 2 publications), the proportion of international co-authorship will be either 100 per cent or 50 per cent, thus much higher than the average for the population which is 35 per cent (Fig 1).

Of more importance for the research question of this paper are the results concerning the age variable. The regression analysis shows that age is negative correlated with international collaboration in all fields—and regardless of academic position. However, the association is only statistically significant (equals to -0.009) for Engineering and technology and Medical and health sciences (-0.008). For the remaining three major fields, the association between age and international collaboration, is weaker, and not significant.

Since the dependent variable is log-transformed, the change caused by a variable is given by the change in B and can be expressed as 100*(e^B^-1) for small values. A beta-value of -0.008 for age means that the average proportion of international co-authored papers per researcher is reduced with 0.8 percent from the initial value per year. As an example, this implies that for researchers within Engineering and technology the proportion of internationally co-authored publications is reduced with 16 percentage points from the age of 40 to the age of 60 (20 years).

Our findings from the regressions do support our hypothesis that age is negatively correlated to international collaboration, but only partly. If we compare the regression results with aggregated statistics from Appendix Table 3 in S1 Appendix which shows the distribution of the average proportion of publication with international collaboration per researcher (indicator B) by major fields, academic position and age groups, we get a more complete picture of the situation. For instance, younger full professors in their 40’s have a higher share of their publications with international collaboration than professors in their 60’s, and the degree of international collaboration is showing a declining pattern for most of the major fields for full professors.

## Discussion & conclusions

The main finding of this study is that there *are* age differences in international collaboration, with the youngest researchers being more international compared to older researchers, but that these differences are not consistent across fields. This concerns both indicators applied: the proportion of researchers involved in international collaboration and the intensity of their international collaboration. Although age differences do not systematically support our main hypothesis, we do *not* find any evidence supporting the alternative hypothesis (that older researchers are more international).

Previous studies on the relation between age and international collaboration have reached different conclusions. There might be several reasons for this. One is that generational differences tend to be relatively small and may depend on particular circumstances of the research systems analysed. Another might be that there are methodological differences in the way international collaboration is analysed. For example, in the study by Kwiek [6] international collaboration was measured using self-reported data on such engagement, with the older generations being more international. However, as discussed by the author, publishing is just one aspect of collaboration, and we believe that it is reasonable to assume that the older generations compared with the younger are more involved in *other* types of international collaborative work such as committee work, network participations, various leadership tasks, etc. Thus, bibliometric data–limited to just one aspect of research collaboration–can give different results on generational differences than e.g., survey data covering all forms of collaboration. Methodological differences also appear in the specific indicators used, age classification systems and personnel analysed. For example, the study by Abramo, D’Angelo & Murgia [32] included the tenured scientific personnel within the Italian research system only (three types of professors). Although their study did not specifically address the age dimension, it is still relevant for comparison with our study, because age is arguably highly correlated to the categories of academic positions that were used. As such, their age-spectrum was different, i.e., narrower, than ours. In order to avoid any type of ecological fallacy in our results, our conclusions are therefore derived from both a regression analysis and from within group analyses combining age and academic position. The importance of taking key compositional characteristics like academic position, productivity and scientific field into account has been firmly clarified in our study, as illustrated in Fig 3 where the aggregate numbers for Proportion of researchers involved in international collaboration (indicator A) corresponds *positively* with age, which is indeed an ecological fallacy as the within-groups associations by academic position are all negative.

As emphasized by Bozeman & Gaughan [51] and Zeng et al. [52], and shown in our study, publication productivity and academic rank are factors which influence on the propensity of international collaboration at the levels of individuals. It is important to note though that our study still has confirmed that even *within* the same academic position, younger staff are more international collaborative than older staff members.

It is, however, none of these factors that matters the most for the inclination to co-author internationally. Rather it is the field of research that is the most decisive factor. Scholars in the humanities have relatively limited engagement in international collaboration, at least in a way which is reflected through bibliometric co-authorship patterns. In the natural sciences, on the other hand such cooperation is very frequent and the large majority of the researchers collaborate with colleagues in other countries.

### Study limitations

We acknowledge that, the generational differences in the propensity to collaborate internationally are not very large. Our dataset does not enable us to provide any detailed (or causal) explanations for this. If younger researchers are indeed more international, one plausible explanation to why the age differences are not bigger is that many publications have both younger and older researchers contributing as co-authors (for example, a PhD-candidate and the full professor serving as supervisor). Thus, the research process and its outcome in terms of publications are often intertwined across generations. Even a more detailed study taking first- and corresponding author position into account would not have been able to overcome this, although we acknowledge that especially for the youngest researchers, the international network of co-authors that they engage with are most likely set up by their older (professor) supervisors.

Most researchers work in groups, but we do not know of any studies addressing the age distributions of individuals in research groups. There is therefore no available empirical evidence about whether research groups have skewed age-distributions, i.e., older researchers are drawn to some groups; while younger are drawn to other groups (similarly to the ‘homophily’ dimension in comparing men and women’s authorship practices, [see 53]). We do not, however, see any *a priori* reasons why this should be the case. It seems more likely to characterize the archetype of a research group as pyramid-like with age increasing towards the top-level of the group. If so, this may help explain the increased international collaboration in young age groups, as they are found at the bottom of the hierarchy with the highest *n*. The younger researchers’ inclination to collaborate internationally was identified regardless of academic position and was found in all major fields–also in terms of publication activity.

While our findings are based on a large dataset, they are limited to just one country: Norway. It is important to emphasize that the proportions of internationally co-authored publications vary greatly across nations [54], generally being much higher in smaller countries than in larger ones. As a small country, the international collaboration rates of Norway will be higher than for many larger nations. Nevertheless, we believe that the patterns identified here may have general relevance because our study is not about the overall level of international collaboration in a country, but the compositional differences *within* a country.

Norway also has a large and increasing volume of international academic staff. This pattern is seen across all fields and academic positions, particularly the lower ranks. The influx of foreigners is generally much higher in engineering and the natural sciences than in the other fields (54). One might assume that the foreigners are more internationally oriented than the Norwegian-born researchers and some of them might continue collaboration with researchers from their home institutions abroad. In turn, this might influence on the findings obtained. We do not have data available to assess to what extent this is the case. However, we note that the generational differences are observed across all fields analysed (except the Social sciences, where age was not statistically significant in the regression analyses), also in the fields where there has been less recruitment from abroad.

### Are younger researchers more international or is research becoming more international?

We see our results as examples of certain global trends in academia where increased international mobility of both students and researchers is the starting point. Numerous studies point at how those who go abroad to study or have research stays sets up future research collaboration from home later on [55–57]; or act as brokers of knowledge [58–60]. Younger generations have entered the research system at a time with different academic norms and practices [cf. 6], especially related to international collaboration. As individuals (who may operate either as lone researchers or as initiators of groups (as members too), the younger researchers may be more cosmopolitan in their research practice than the older staff. This again might be linked to differences in the socio-cultural factors influencing the researchers when being trained into the academic profession. The older researchers were trained at a time when international collaboration was much less common than today, which still to some extent influence their collaboration practice. This harmonizes with theories of global identity/citizenship [e.g., 25].

Another explanation is less focusing on the researchers themselves, but on the research system. Globally, research teams are getting bigger, partly due to large scale infrastructure involving many nations [e.g., 61], e.g. the developing of ‘big science’ [e.g., 62], and the increasing complexity of science, which necessitates multidisciplinary and highly specialized approaches, thus leading to an ever expanding increase in the numbers of scientists, institutions and countries that work together in research projects [63–66]. Because countries tend to specialize in different scientific disciplines [e.g., 67], going international is often the most feasible option to gain necessary complementary expertise.

In the generation of global ubiquity [26], transaction costs have become low [62]. As such, scientists’ international involvement is driven by ‘evolution’ equal to ‘choice’, due to the increased complexity of science. Related to this is the fact that many funding agencies use their schemes to steer researchers towards more cross-national cooperation [e.g., 68]. We believe that since more and more research is conducted in informal or formal *teams* (or ‘research groups’), working across borders, it will on an aggregate level move the younger researchers more than the older researchers towards being international on our two indicators (proportion of researchers doing international research, and the intensity of their international collaboration).

## Supporting information

S1 Appendix (DOCX)

## References

[1] Adams J. The rise of research networks. Nature. 2012;490(7420):335–6. doi: 10.1038/490335a 23075965

[2] Wagner CS, Whetsell TA, Leydesdorff L. Growth of international collaboration in science: revisiting six specialties. Scientometrics. 2017;110(3):1633–52.

[3] Abramo G, D’Angelo CA, Solazzi M. The relationship between scientists’ research performance and the degree of internationalization of their research. Scientometrics. 2011;86(3):629–43.

[4] Kwiek M. Academic generations and academic work: patterns of attitudes, behaviors, and research productivity of Polish academics after 1989. Studies in Higher Education. 2015;40(8):1354–76.

[5] Abramo G, D’Angelo CA, & Murgia G. (2015). The combined effects of age and seniority on research performance of full professors. Science and Public Policy. 2015; 43(3): 301–319.

[6] Kwiek M. International Research Collaboration and International Research Orientation: Comparative Findings About European Academics. Journal of Studies in International Education. 2018;22(2):136–60.

[7] Kyvik S, Aksnes DW. Explaining the increase in publication productivity among academic staff: a generational perspective. Studies in Higher Education. 2015;40(8):1438–53.

[8] Ochsner M, Kulczycki E, Gedutis A. The Diversity of European Research Evaluation Systems. STI 2018 Conference Proceedings Leiden: CTWS, Leiden University; 2018.

[9] Breugelmans JG, Roberge G, Tippett C, Durning M, Struck DB, Makanga MM. Scientific impact increases when researchers publish in open access and international collaboration: A bibliometric analysis on poverty-related disease papers. Plos One. 2018;13(9). doi: 10.1371/journal.pone.0203156 30231044 PMC6145557

[10] Abramo G, D’Angelo CA, Di Costa F. Research collaboration and productivity: is there correlation? High Educ. 2009;57(2):155–71.

[11] Barjak F, Robinson S. International collaboration, mobility and team diversity in the life sciences: Impact on research performance. Social Geography. 2008;3:23–6.

[12] Katz SJ, Martin BR. What is research collaboration? Research Policy. 1997;26(1):1–18.

[13] Aksnes DW, Sivertsen G. A Criteria-based Assessment of the Coverage of Scopus and Web of Science. Journal of Data and Information Science. 2019;4(1):1–21.

[14] Barjak F. Research productivity in the internet era. Scientometrics. 2006;68(3):343–60.

[15] Gingras Y, Lariviere V, Macaluso B, Robitaille JP. The Effects of Aging on Researchers’ Publication and Citation Patterns. Plos One. 2008;3(12). doi: 10.1371/journal.pone.0004048 19112502 PMC2603321

[16] Gonzalez-Brambila C, Veloso FM. The determinants of research output and impact: A study of Mexican researchers. Research Policy. 2007;36(7):1035–51.

[17] Merton RK, Zuckerman H. Age, aging and age structure in science. In: Merton RK, editor. The Sociology of Science. Chicago: The University of Chicago Press; 1973.

[18] Aksnes DW, Rorstad K, Piro F, Sivertsen G, editors. Age and scientific performance. A large-scale study of Norwegian scientists. ISSI 2011; 2011; Durban, South Africa.

[19] Sugimoto CR, Sugimoto TJ, Tsou A, Milojevic S, Lariviere V. Age stratification and cohort effects in scholarly communication: a study of social sciences. Scientometrics. 2016;109(2):997–1016.

[20] Liu L, Wang Y, Sinatra R, Giles CL, Song C, Wang D. Hot streaks in artistic, cultural, and scientific careers. Nature. 2018; 559(7714), 396–399. doi: 10.1038/s41586-018-0315-8 29995850

[21] Jones B, Reedy EJ, Weinberg BA. Age and scientific genius (No. w19866). Cambridge: National Bureau of Economic Research; 2014.

[22] Lee SJ, Jung JS. Work experiences and knowledge transfer among Korean academics: focusing on generational differences. Studies in Higher Education. 2018;43(11):2033–58.

[23] Jones BF, Weinberg BA. Age dynamics in scientific creativity. Proceedings of the National Academy of Sciences of the United States of America. 2011;108(47):18910–4. doi: 10.1073/pnas.1102895108 22065777 PMC3223465

[24] Packalen M, Bhattacharya J. Age and the Trying Out of New Ideas. Journal of Human Capital. 2019;13(2):341–73. doi: 10.1086/703160 31435457 PMC6703833

[25] Norris P. Global governance and cosmopolitan citizens. In Nye JS, Donahue JD (eds.) Governance in a Globalizing World. Washington, DC: Brookings Institution Press. 2000, pp. 155–177.

[26] Stohl C, Ganesh S. Generating Globalization. In Mumby DK, Putnam LL (eds.). The Sage Handbook of Organizational Communication (3rd ed., pp. 717–741). Newbury Park, CA: Sage Publications.

[27] Eduan W. Influence of study abroad factors on international research collaboration: evidence from higher education academics in sub-Saharan Africa. Studies in Higher Education. 2019;44(4):774–85.

[28] Norris EM, Gillespie J. How Study Abroad Shapes Global Careers Evidence From the United States. Journal of Studies in International Education. 2009;13(3):382–97.

[29] Kyvik S. Age and scientific productivity. differences between fields of learning. High Educ. 1990;19:37–55.

[30] Jeong S, Choi JY, Kim J. The determinants of research collaboration modes: exploring the effects of research and researcher characteristics on co-authorship. Scientometrics. 2011;89(3):967–83.

[31] Hinnant CC, Stvilia B, Wu SH, Worrall A, Burnett G, Burnett K, et al. Author-team diversity and the impact of scientific publications: Evidence from physics research at a national science lab. Library & Information Science Research. 2012;34(4):249–57.

[32] Abramo G, D’Angelo CA, Murgia G. Variation in research collaboration patterns across academic ranks. Scientometrics. 2014;98(3):2275–94.

[33] Kawaguchi D, Kondo A, Saito K. Researchers’ career transitions over the life cycle. Scientometrics. 2016;109(3):1435–54.

[34] Jeong S, Choi JY, Kim JY. On the drivers of international collaboration: The impact of informal communication, motivation, and research resources. Science and Public Policy. 2014;41(4):520–31.

[35] Ebadi A, Schiffauerova A. How to Receive More Funding for Your Research? Get Connected to the Right People! Plos One. 2015;10(7):19. doi: 10.1371/journal.pone.0133061 26222598 PMC4519253

[36] Daniels RJ. A generation at risk: Young investigators and the future of the biomedical workforce. Proceedings of the National Academy of Sciences of the United States of America. 2015;112(2):313–8. doi: 10.1073/pnas.1418761112 25561560 PMC4299207

[37] Ebadi A, Schiffauerova A. How to become an important player in scientific collaboration networks? Journal of Informetrics. 2015;9(4):809–25.

[38] Abramo G, D’Angelo CA, Di Costa F. Research productivity: Are higher academic ranks more productive than lower ones? Scientometrics. 2011;88(3):915–28.

[39] Wagner CS, Leydesdorff L. Network structure, self-organization, and the growth of international collaboration in science. Research Policy. 2005; 34(10), 1608–1618.

[40] Bozeman B, Corley E. Scientists’ collaboration strategies: implications for scientific and technical human capital. Research Policy. 2004; 33(4), 599–616.

[41] Kyvik S, Olsen TB. Does the aging of tenured academic staff affect the research performance of universities? Scientometrics. 2008;76(3):439–55.

[42] Kyvik S, Reymert I. Research collaboration in groups and networks: differences across academic fields. Scientometrics. 2017;113(2):951–67. doi: 10.1007/s11192-017-2497-5 29081555 PMC5640763

[43] Sivertsen G. The Norwegian Model in Norway. Journal of Data and Information Science. 2018;3(4):3–19.

[44] Aksnes DW. When different persons have an identical author name, How frequent are homonyms? Journal of the American Society for Information Science and Technology. 2008; 59(5), 838–841.

[45] National Science Board. Science and Engineering Indicators 2018. Alexandria, VA: National Science Foundation; 2018.

[46] Aksnes DW, Frolich N, Slipersaeter S. Science policy and the driving forces behind the internationalisation of science: the case of Norway. Science and Public Policy. 2008;35(6):445–57.

[47] Mayer SJ, Rathmann JMK. How does research productivity relate to gender? Analyzing gender differences for multiple publication dimensions. Scientometrics. 2018;117(3):1663–93.

[48] Aksnes DW, Piro FN, Rorstad K. Gender gaps in international research collaboration: a bibliometric approach. Scientometrics. 2019;120(2):747–74.

[49] Pearl J. Comment: Understanding Simpson’s Paradox. American Statistician. 2014;68(1):8–13.

[50] O’Brien RM. A Caution Regarding Rules of Thumb for Variance Inflation Factors. Quality & Quantity. 2007; 41:673–690. doi: 10.1007/s11135-006-9018-6

[51] Bozeman B, Gaughan M. How do men and women differ in research collaborations? An analysis of the collaborative motives and strategies of academic researchers. Research Policy. 2011;40(10):1393–402.

[52] Zeng XHT, Duch J, Sales-Pardo M, Moreira JAG, Radicchi F, Ribeiro HV, et al. Differences in Collaboration Patterns across Discipline, Career Stage, and Gender. Plos Biology. 2016;14(11). 10.1371/journal.pbio.1002573 PMC5096717 27814355

[53] Gallivan M, Ahuja M. Co-authorship, Homophily, and Scholarly Influence in Information Systems Research. Journal of the Association for Information Systems. 2015;16(12):980–1015.

[54] Frølich N, Reiling RB, Gunnes H, Mangset M, Orupabo J, Ulvestad MES, et al. Attraktive akademiske karrierer? Søkning, rekruttering og mobilitet i UH-sektoren. NIFU Nordisk institutt for studier av innovasjon, forskning og utdanning. Rapport 2019:10. 2019. Retrieved from http://hdl.handle.net/11250/2608244.

[55] Andujar I, Canibano C, Fernandez-Zubieta A. International Stays Abroad, Collaborations and the Return of Spanish Researchers. Science Technology and Society. 2015;20(3):322–48.

[56] Jonkers K, Tijssen R. Chinese researchers returning home: Impacts of international mobility on research collaboration and scientific productivity. Scientometrics. 2008;77(2):309–33.

[57] Marmolejo-Leyva R, Perez-Angon MA, Russell JM. Mobility and International Collaboration: Case of the Mexican Scientific Diaspora. Plos One. 2015;10(6). doi: 10.1371/journal.pone.0126720 26047501 PMC4457895

[58] Fontes M, Videira P, Calapezy T. The Impact of Long-term Scientific Mobility on the Creation of Persistent Knowledge Networks. Mobilities. 2013;8(3):440–65.

[59] Baruffaldi SH, Landoni P. Return mobility and scientific productivity of researchers working abroad: The role of home country linkages. Research Policy. 2012;41(9):1655–65.

[60] Murakami Y. Influences of return migration on international collaborative research networks: cases of Japanese scientists returning from the US. Journal of Technology Transfer. 2014;39(4):616–34.

[61] D’Ippolito B, Ruling CC. Research collaboration in Large Scale Research Infrastructures: Collaboration types and policy implications. Research Policy. 2019;48(5):1282–96.

[62] Hand E. ‘Big science’ spurs collaborative trend. Nature. 2010;463:282. doi: 10.1038/463282a 20090723

[63] Porter A, Rafols I. Is science becoming more interdisciplinary? Measuring and mapping six research fields over time. Scientometrics. 2009;81(3):719–745.

[64] Qiu L. A study of interdisciplinary research collaboration. Research Evaluation. 1992;2(3):169–175.

[65] Van Noorden R. Interdisciplinary Research by the Numbers. Nature. 2015;525:306–307. doi: 10.1038/525306a 26381967

[66] Adams JD, Black GC, Clemmons JR, Stephan PE. Scientific teams and institutional collaborations: Evidence from US universities, 1981–1999. Research Policy. 2005;34(3):259–85.

[67] Aksnes DW, van Leeuwen TN, Sivertsen G. The effect of booming countries on changes in the relative specialization index (RSI) on country level. Scientometrics. 2014;101:1391–1401.

[68] Mattsson P, Laget P, Vindefjard AN, Sundberg CJ. What do European research collaboration 994 networks in life sciences look like? Research Evaluation. 2010;19(5):373–84.

