# Supplementary material for: Generational differences in international research collaboration: A bibliometric study of Norwegian University staff
Source: PLoS One. 2021 Nov 29;16(11):e0260239. doi: 10.1371/journal.pone.0260239 (PMC8629247; doi:10.1371/journal.pone.0260239)
Supplement: S1 Appendix — (DOCX) [file pone.0260239.s001.docx]

**S1 Appendix**

Appendix table 1. Distribution of the number of publications by academic position, major fields and age groups (N=43,641)

| *Academic position/major fields* | *Below 30 years* | *30-39 years* | *40-49 years* | *50-59 years* | *Over 60 years* | *Total* |
| --- | --- | --- | --- | --- | --- | --- |
| Full professors |  | 802 | 6,829 | 9,969 | 8,251 | 25,851 |
| *Humanities* |  | *127* | *489* | *721* | *714* | *2,051* |
| *Social sciences* |  | *125* | *552* | *1,013* | *1,038* | *2,728* |
| *Natural sciences* |  | *256* | *2,364* | *3,265* | *2,338* | *8,223* |
| *Engineering and technology* |  | *216* | *1,643* | *1,585* | *1,120* | *4,564* |
| *Medical and health sciences* |  | *78* | *1,781* | *3,385* | *3,041* | *8,285* |
| Associate professors | 31 | 2,376 | 3,731 | 1,455 | 788 | 8,381 |
| *Humanities* |  | *178* | *439* | *186* | *117* | *920* |
| *Social sciences* |  | *354* | *691* | *350* | *174* | *1,569* |
| *Natural sciences* | *2* | *572* | *952* | *214* | *112* | *1,852* |
| *Engineering and technology* | *29* | *757* | *495* | *154* | *130* | *1,565* |
| *Medical and health sciences* |  | *515* | *1,154* | *551* | *255* | *2,475* |
| Postdocs | 179 | 3,393 | 657 | 85 | 56 | 4,370 |
| *Humanities* |  | *140* | *74* | *5* |  | *219* |
| *Social sciences* |  | *274* | *112* | *5* |  | *391* |
| *Natural sciences* | *43* | *1,579* | *189* | *13* | *15* | *1,839* |
| *Engineering and technology* | *99* | *627* | *16* |  | *2* | *744* |
| *Medical and health sciences* | *37* | *773* | *266* | *62* | *39* | *1177* |
| PhD-candidates | 2,390 | 2,160 | 422 | 58 | 9 | 5,039 |
| *Humanities* | *34* | *191* | *35* | *4* |  | *264* |
| *Social sciences* | *95* | *188* | *72* | *17* | *6* | *378* |
| *Natural sciences* | *1,136* | *744* | *26* | *8* | *3* | *1,917* |
| *Engineering and technology* | *809* | *386* | *41* | *8* |  | *1,244* |
| *Medical and health sciences* | *316* | *651* | *248* | *21* |  | *1,236* |
| **Total** | **2,600** | **8,731** | **11,639** | **11,567** | **9,104** | **43,641** |

**Appendix table 2. Distribution of the proportion of researchers involved in international collaboration by major fields, academic position, and age group (indicator A)**

| *Academic position/major fields* | Below 30 years | 30-39 years | 40-49 years | 50-59 years | Over 60 years | Total |
| --- | --- | --- | --- | --- | --- | --- |
| Humanities | 10 % | 28 % | 33 % | 36 % | 31 % | 32 % |
| *Full professors* |  | *80 %* | *40 %* | *44 %* | *29 %* | *38 %* |
| *Associate professors* |  | *36 %* | *33 %* | *22 %* | *38 %* | *32 %* |
| *Postdocs* |  | *28 %* | *28 %* | *0 %* |  | *27 %* |
| *PhD-candidates* | *10 %* | *18 %* | *12 %* | *0 %* |  | *15 %* |
| Social sciences | 16 % | 35 % | 40 % | 46 % | 42 % | 40 % |
| *Full professors* |  | *46 %* | *57 %* | *57 %* | *47 %* | *53 %* |
| *Associate professors* |  | *49 %* | *36 %* | *28 %* | *26 %* | *35 %* |
| *Postdocs* |  | *48 %* | *35 %* | *50 %* |  | *45 %* |
| *PhD-candidates* | *16 %* | *18 %* | *21 %* | *29 %* | *33 %* | *19 %* |
| Natural sciences | 66 % | 78 % | 89 % | 88 % | 89 % | 81 % |
| *Full professors* |  | *100 %* | *98 %* | *93 %* | *89 %* | *93 %* |
| *Associate professors* | *100 %* | *80 %* | *87 %* | *67 %* | *87 %* | *81 %* |
| *Postdocs* | *75 %* | *84 %* | *71 %* | *100 %* | *100 %* | *83 %* |
| *PhD-candidates* | *65 %* | *67 %* | *13 %* | *67 %* | *100 %* | *65 %* |
| Engineering and technology | 50 % | 62 % | 80 % | 75 % | 72 % | 65 % |
| *Full professors* |  | *100 %* | *91 %* | *81 %* | *81 %* | *85 %* |
| *Associate professors* | *50 %* | *74 %* | *70 %* | *53 %* | *29 %* | *64 %* |
| *Postdocs* | *38 %* | *75 %* | *75 %* |  | *0 %* | *71 %* |
| *PhD-candidates* | *51 %* | *40 %* | *40 %* | *100 %* |  | *47 %* |
| Medicine and health sciences | 63 % | 65 % | 74 % | 84 % | 80 % | 73 % |
| *Full professors* |  | *67 %* | *92 %* | *91 %* | *82 %* | *88 %* |
| *Associate professors* |  | *81 %* | *72 %* | *75 %* | *67 %* | *74 %* |
| *Postdocs* | *70 %* | *77 %* | *89 %* | *60 %* | *100 %* | *79 %* |
| *PhD-candidates* | *63 %* | *53 %* | *47 %* | *31 %* |  | *54 %* |
| Total | 55 % | 59 % | 63 % | 67 % | 63 % | 62 % |

**Appendix table 3. Distribution of the average proportion of publications with international collaboration per researcher (per cent) by major fields, academic position and age group (indicator B)**

| Fields/positions | Below 30 years | 30-39 years | 40-49 years | 50-59 years | Over 60 years | Total/average |
| --- | --- | --- | --- | --- | --- | --- |
| Humanities | 10% | 12% | 13% | 13% | 12% | 12% |
| *Full professors* |  | *25 %* | *16 %* | *16 %* | *10 %* | *14 %* |
| *Associate professors* |  | *12 %* | *14 %* | *9 %* | *17 %* | *13 %* |
| *Postdocs* |  | *11 %* | *11 %* | *0 %* |  | *11 %* |
| *PhD-candidates* | *10 %* | *10 %* | *2 %* | *0 %* |  | *9 %* |
| Social sciences | 12% | 17% | 16% | 18% | 18% | 17% |
| *Full professors* |  | *17 %* | *25 %* | *21 %* | *18 %* | *21 %* |
| *Associate professors* |  | *25 %* | *13 %* | *13 %* | *14 %* | *16 %* |
| *Postdocs* |  | *18 %* | *10 %* | *17 %* |  | *16 %* |
| *PhD-candidates* | *12 %* | *10 %* | *13 %* | *9 %* | *33 %* | *11 %* |
| Natural sciences | 51% | 59% | 57% | 57% | 57% | 56% |
| *Full professors* |  | *71 %* | *64 %* | *60 %* | *57 %* | *60 %* |
| *Associate professors* | *100 %* | *53 %* | *54 %* | *42 %* | *51 %* | *51 %* |
| *Postdocs* | *43 %* | *63 %* | *49 %* | *100 %* | *27 %* | *61 %* |
| *PhD-candidates* | *51 %* | *53 %* | *7 %* | *58 %* | *33 %* | *51 %* |
| Engineering and technology | 29% | 33% | 36% | 33 % | 31 % | 32 % |
| *Full professors* |  | *59 %* | *41 %* | *37 %* | *34 %* | *38 %* |
| *Associate professors* | *13 %* | *31 %* | *29 %* | *21 %* | *14 %* | *27 %* |
| *Postdocs* | *14 %* | *43 %* | *58 %* |  | *0 %* | *41 %* |
| *PhD-candidates* | *29 %* | *22 %* | *23 %* | *64 %* |  | *27 %* |
| Medical and health sciences | 46 % | 44 % | 42 % | 44 % | 41 % | 43 % |
| *Full professors* |  | *40 %* | *52 %* | *50 %* | *41 %* | *47 %* |
| *Associate professors* |  | *43 %* | *39 %* | *32 %* | *39 %* | *38 %* |
| *Postdocs* | *46 %* | *52 %* | *50 %* | *29 %* | *20 %* | *50 %* |
| *PhD-candidates* | *46 %* | *39 %* | *31 %* | *27 %* |  | *39 %* |
| Total/average | 39 % | 38 % | 34 % | 34 % | 32 % | 35 % |
